# Effects of Combined Physical and Cognitive Interventions in Older Adults with Dementia: A Meta-Analysis

**DOI:** 10.1093/geroni/igaf122.2852

**Published:** 2025-12-31

**Authors:** Dandan Xue, Yanqiu Hu, Rui Zhang

**Affiliations:** Gongli Hospital of Shanghai Pudong New Area, Shanghai, Shanghai, China (People’s Republic); Eye & ENT Hospital, Shanghai, Shanghai, China (People’s Republic); Naval Medical University, Shanghai, Shanghai, China (People’s Republic)

## Abstract

The effects of combined physical and cognitive interventions in persons with dementia remains inconsistent. This review aims to compare the effects of combined interventions with those of each intervention alone on cognitive, physical, psychological, functional, and health-related quality of life outcomes for persons with dementia. Searches were conducted across 8 English and Chinese databases from their inception dates to September 10, 2024. Quality appraisal was conducted using Cochrane’s risk of bias tool. Random-effects models were employed for meta-analysis, and subgroup analyses and meta-regression were performed to explore potential moderators. A total of 23 studies involving 1,716 participants were included. Combined interventions significantly improved global cognition [SMD = 0.65, 95%CI (0.35, 0.95), p < 0.001], processing speed [SMD = 0.33, 95%CI (0.03, 0.63), p = 0.03], functional mobility [SMD = 0.85, 95% CI (0.18–1.53), p = 0.01], strength [SMD = 0.95, 95% CI (0.28, 1.61), p = 0.005], depression [SMD = -1.04, 95% CI (-1.73, -0.36), p = 0.003], and health-related quality of life [SMD = 0.71; 95% CI (0.24, 1.18), p = 0.003] compared to active or passive controls. Combining interventions didn’t provide better overall benefits than using either intervention alone. Further analysis showed that the effectiveness of combined interventions on global cognition depended on the age of the participants. In conclusion, combined interventions were beneficial for persons with dementia, and these interventions are recommended to be integrated into dementia management protocols. Future multi-arm randomized controlled trials with long-term follow-ups are warranted.

